# Tendência Temporal no Implante Percutâneo de Bioprótese Aórtica: Análise de 10 Anos do Registro TAVIDOR

**DOI:** 10.36660/abc.20230467

**Published:** 2024-05-23

**Authors:** Vinicius Esteves, Pedro Beraldo de Andrade, Cleverson Neves Zukowski, Edmur Araujo, Cristiano Guedes Bezerra, Adriano Dourado Oliveira, Eduardo Pessoa de Melo, Gustavo Gama, Rodrigo Cantarelli, Luiz Alberto Piva e Mattos, Angelo Tedeschi, Vitor Alves Loures, Vitor Vahle, Guilherme Barreto Gameiro Silva, Miguel Antonio Neves Rati, Augusto Celso Lopes, Nilson de Moura Fé, Gustavo Alves, Sérgio Costa Tavares, Sergio Kreimer, Marden Tebet, Felipe Maia, Maurício Sales de Oliveira, Alberto Fonseca, Angelina Camiletti, Denilson Campos de Albuquerque, Olga Ferreira de Souza

**Affiliations:** 1 Rede D’Or São Luiz São Paulo SP Brasil Rede D’Or São Luiz, São Paulo, SP – Brasil; 2 Santa Casa de Misericórdia de Marília Marília SP Brasil Santa Casa de Misericórdia de Marília – Cardiologia Invasiva, Marília, SP – Brasil; 3 Hospital Copa D’Or Rio de Janeiro RJ Brasil Hospital Copa D’Or, Rio de Janeiro, RJ – Brasil; 4 Hospital Quinta D’Or Rio de Janeiro RJ Brasil Hospital Quinta D’Or, Rio de Janeiro, RJ – Brasil; 5 Hospital do Coração do Brasil Brasília RJ Brasil Hospital do Coração do Brasil, Brasília, RJ – Brasil; 6 Universidade Federal da Bahia Salvador BA Brasil Universidade Federal da Bahia – Hemodinâmica e Cardiologia Intervencionista, Salvador, BA – Brasil; 7 Hospital Santa Izabel Salvador BA Brasil Hospital Santa Izabel, Salvador, BA – Brasil; 8 Hospital Esperança Recife PE Brasil Hospital Esperança, Recife, PE – Brasil; 9 UDI Hospital São Luís MA Brasil UDI Hospital, São Luís, MA – Brasil; 10 Hospital Memorial São José Recife PE Brasil Hospital Memorial São José, Recife, PE – Brasil; 11 Rede D’Or São Luiz Rio de Janeiro RJ Brasil Rede D’Or São Luiz, Rio de Janeiro, RJ – Brasil; 12 Hospital São Luiz Anália Franco São Paulo SP Brasil Hospital São Luiz Anália Franco, São Paulo, SP – Brasil; 13 Hospital São Lucas Aracaju SE Brasil Hospital São Lucas, Aracaju, SE – Brasil; 14 Hospital Santa Cruz Curitiba PR Brasil Hospital Santa Cruz, Curitiba, PR – Brasil; 15 Hospital Barra D’Or Rio de Janeiro RJ Brasil Hospital Barra D’Or, Rio de Janeiro, RJ – Brasil; 16 Hospital Monte Klinikum Fortaleza CE Brasil Hospital Monte Klinikum, Fortaleza, CE – Brasil; 17 Hospital São Carlos Fortaleza CE Brasil Hospital São Carlos, Fortaleza, CE – Brasil; 18 Hospital e Maternidade Brasil Santo André SP Brasil Hospital e Maternidade Brasil, Santo André, SP – Brasil; 19 Universidade do Estado do Rio de Janeiro Rio de Janeiro RJ Brasil Universidade do Estado do Rio de Janeiro, Rio de Janeiro, RJ – Brasil

**Keywords:** Estenose da Valva Aórtica, Substituição da Valva Aórtica Transcateter, Análise Espaço-Temporal, Idoso

## Abstract

**Fundamento:**

O implante percutâneo de bioprótese valvar aórtica (TAVI) consolidou-se como opção terapêutica da estenose aórtica de grau importante. Dados sobre as características evolutivas dos procedimentos e dos resultados obtidos com a técnica ao longo da última década, em escala nacional, são desconhecidos.

**Objetivos:**

Analisar a tendência temporal referente ao perfil demográfico, características dos procedimentos e desfechos hospitalares de pacientes submetidos a TAVI na Rede D’Or São Luiz.

**Métodos:**

Registro observacional envolvendo 29 instituições nacionais. Comparou-se características dos procedimentos realizados de 2012 a 2017 (Grupo 1) e de 2018 a 2023 (Grupo 2). Foram considerados significantes os resultados com valor de p < 0,05.

**Resultados:**

Foram analisados 661 casos, 95 pertencentes ao Grupo 1 e 566 ao Grupo 2. A média de idade foi 81,1 anos. Observou-se no Grupo 1 maior prevalência de pacientes em classe funcional III ou IV e escore de risco > 8%. Foi mais frequente o emprego de anestesia geral, monitorização ecocardiográfica transesofágica e via de acesso por dissecção. Maior taxa de sucesso do procedimento (95,4% versus 89,5%; p = 0,018) foi aferida em implantes efetivados a partir de 2018, assim como menor mortalidade (3,9% versus 11,6%; p = 0,004) e necessidade de marcapasso definitivo (8,5% versus 17,9%; p = 0,008).

**Conclusões:**

A análise temporal de 10 anos do Registro TAVIDOR demonstra uma queda na complexidade clínica dos pacientes. Além disso, o avanço para técnicas de implante minimalistas, somadas à evolução tecnológica dos dispositivos, podem ter contribuído para desfechos favoráveis dentre aqueles cujo implante ocorreu no último quinquênio.

## Graphical abstract

**Figura Central:**
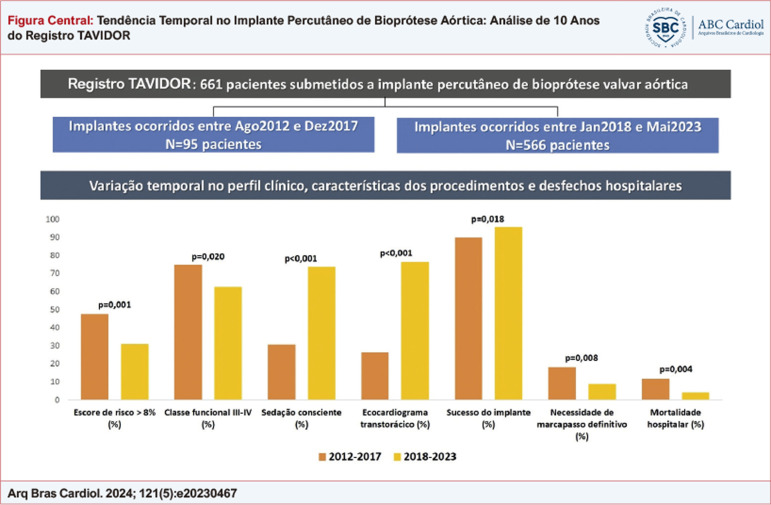
: Tendência Temporal no Implante Percutâneo de Bioprótese Aórtica: Análise de 10 Anos do Registro TAVIDOR Variação temporal no perfil clínico, características dos procedimentos e desfechos hospitalares de pacientes submetidos a implante percutâneo de bioprótese valvar aórtica. NYHA: New York Heart Association; STS: Society of Thoracic Surgeons.

## Introdução

O implante percutâneo de bioprótese valvar aórtica (TAVI) consolidou-se como estratégia preferencial na abordagem da estenose aórtica de grau importante, sintomática em pacientes com idade maior ou igual a 70 anos, portadores de risco cirúrgico proibitivo, contraindicações à cirurgia convencional ou com relevante fragilidade.^1^

Na trajetória exitosa dessa modalidade terapêutica, foi determinante que os dispositivos de primeira geração tivessem sua eficácia e segurança inicialmente avaliados em pacientes inoperáveis,^2^ e que a consequente transição para indivíduos com menor perfil de complexidade ocorresse simultaneamente aos avanços no desenho das próteses, incorporação da saia de vedação externa, possibilidade de recaptura, redução de perfil e do calibre dos introdutores, bem como aprimoramentos na técnica de implante, traduzindo-se em menores taxas de regurgitação paravalvar, necessidade de marcapasso definitivo, acidente vascular encefálico e complicações vasculares.^3-6^

Após a primeira descrição de TAVI em humanos com um dispositivo balão expansível no ano de 2002, por Cribier et al.,^7^ e com uma prótese autoexpansível em 2005 por Grube et al.,^8^ registros internacionais passaram a reportar a reprodutibilidade da técnica na obtenção de resultados de eficácia e segurança favoráveis.^9,10^A experiência inicial no Brasil data de 2008,^11^ e desde então, dados acerca dos resultados obtidos no país advêm de publicações geradas a partir da análise do Registro Brasileiro de Implante por Cateter de Bioprótese Valvar Aórtica, sendo este multicêntrico, de participação voluntária e gerenciado pela Sociedade Brasileira de Hemodinâmica e Cardiologia Intervencionista.^12,13^ Entretanto, informações sobre as características evolutivas dos pacientes e dos procedimentos, bem como dos resultados obtidos com a técnica ao longo da última década, em escala nacional, são desconhecidos.

## Objetivos

Analisar a tendência temporal referente ao perfil demográfico, características dos procedimentos e desfechos hospitalares de pacientes submetidos a TAVI no período de 2012 a 2017, comparando-os aos de pacientes tratados entre 2018 e maio de 2023, perfazendo assim dois intervalos de tempo equiparáveis entre os grupos.

## Métodos

### Desenho e população do estudo

Trata-se de um registro observacional, multicêntrico, envolvendo 29 instituições nacionais localizadas nos estados de São Paulo (9), Rio de Janeiro (7), Pernambuco (4), Bahia (3), Ceará (1), Maranhão (1), Sergipe (1), Paraná (1) e Distrito Federal (2), de temporalidade mista, incluindo retrospectivamente pacientes submetidos a TAVI no período de agosto de 2012 a dezembro de 2019, e de forma prospectiva aqueles cujo procedimento foi realizado a partir de janeiro de 2020.

Foram incluídos pacientes portadores de estenose aórtica de grau importante, com idade ≥ 60 anos, submetidos a TAVI após a obtenção de termo de consentimento livre e esclarecido. Foram excluídos do registro pacientes que não realizaram estudo angiotomográfico antecedendo o procedimento.

O registro foi aprovado pelo Comitê de Ética em Pesquisa do Instituto D’Or de Pesquisa e Ensino, e segue as recomendações da Organização Mundial de Saúde, da Declaração dos Direitos de Helsinque e da Resolução 466/2012 do Conselho Nacional de Saúde.

### Procedimentos do estudo

A decisão pela realização de TAVI, bem como a escolha do disposto utilizado, deu-se após reunião de equipe multidisciplinar envolvendo cardiologista clínico, cardiologista intervencionista e cirurgião cardíaco (*heart team*). Procedimentos inerentes ao implante e cuidados até a alta hospitalar seguiram a rotina institucional de cada centro participante. O protocolo do registro TAVIDOR determina o acompanhamento, por meio de contato telefônico, eletrônico (e-mail) ou visita presencial ambulatorial aos 30 dias, 6 e 12 meses, e a seguir anualmente por um período de 5 anos, sendo os pacientes ou familiares inquiridos com relação a sintomas, medicações em uso, exames laboratoriais e desfechos clínicos como hospitalizações e eventos seguindo os critérios do Valve Academic Research Consortium 3 (VARC-3).^14^ O critério adotado para a divisão dos grupos foi a criação de dois extratos populacionais perfazendo aproximadamente 5 anos de observação cada.

### Análise estatística

Os dados foram extraídos da plataforma REDCap, utilizada pelos centros para inserção *on-line* das informações referentes aos pacientes e procedimentos. A análise estatística foi realizada com o programa de software SPSS, versão 17.0 (SPSS Inc., Chicago, IL, EUA). As variáveis categóricas foram descritas pela sua frequência e as variáveis contínuas pela média e desvio padrão ou mediana e intervalo interquartil, de acordo com o padrão de distribuição, avaliado pelo teste de Kolmogorov-Smirnov. Na análise univariada, as variáveis categóricas foram comparadas pelo teste de qui-quadrado e as contínuas comparadas com o teste t de Student ou teste exato de Fisher. Foram considerados estatisticamente significantes os resultados com valor de p < 0,05.

## Resultados

**Figura 1:**
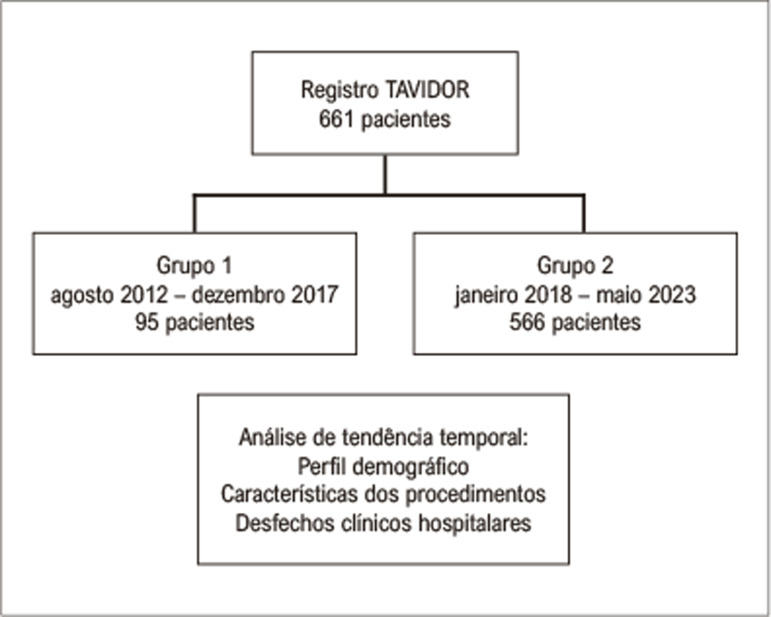
***–*** Fluxograma da análise temporal de pacientes submetidos ao implante percutâneo de bioprótese valvar aórtica.

A Figura 1 ilustra o fluxograma dos pacientes submetidos a TAVI na presente análise. No período de agosto de 2012 a maio de 2023 foram realizados 661 procedimentos, dos quais 95 compreendidos até dezembro de 2017 (Grupo 1) e 566 a partir de janeiro de 2018 (Grupo 2).

A média de idade dos pacientes foi de 81,1 anos, com predomínio de etiologia degenerativa da doença valvar (92,4%) e elevada prevalência de doença aterosclerótica coronariana concomitante (38,5%), insuficiência renal crônica (30,7%) e doença arterial periférica (24,7%). Observou-se no Grupo 1 maior percentual de mulheres, classe funcional III ou IV da New York Heart Association (NYHA) e escore de risco da Society of Thoracic Surgeons (STS) > 8%, quando comparado ao Grupo 2 (Tabela 1).

**Tabela 1:** – Características basais da população geral e por período de inclusão

Características clínicas	Total 2012 – 2023 n=661	Grupo 1 2012 – 2017 n=95	Grupo 2 2018 – 2023 n=566	Valor de p*
Sexo feminino	324 (49,0)	57 (60,0)	267 (47,2)	0,022
Sexo masculino	337 (51,0)	38 (40,0)	299 (52,8)	0,022
Idade, anos	81,1 ± 7,1	81,3 ± 6,6	81,0 ± 7,6	0,748
Hipertensão arterial	560 (84,7)	78 (82,1)	482 (85,2)	0,444
Diabetes mellitus	234 (35,4)	33 (34,7)	201 (35,5)	0,884
Dislipidemia	412 (62,3)	52 (54,7)	360 (63,6)	0,099
Tabagismo	36 (5,4)	2 (2,1)	34 (6,0)	0,121
Insuficiência renal crônica	203 (30,7)	28 (29,5)	175 (30,9)	0,778
Hemodiálise	18 (2,7)	1 (1,1)	17 (3,0)	0,280
Doença arterial periférica	163 (24,7)	24 (25,3)	139 (24,6)	0,883
Doença carotídea	35 (5,3)	3 (3,2)	32 (5,7)	0,315
DPOC	98 (14,8)	15 (15,8)	83 (14,7)	0,775
Fibrilação atrial	125 (20,0)	12 (13,0)	113 (21,2)	0,118
IAM prévio	91 (13,8)	13 (13,7)	78 (13,8)	0,980
AVE prévio	44 (6,7)	4 (4,2)	40 (7,1)	0,301
ICP prévia	184 (27,8)	23 (24,2)	161 (28,4)	0,394
RM prévia	71 (10,7)	14 (14,7)	57 (10,1)	0,174
Troca valvar aórtica prévia	27 (4,1)	2 (2,1)	25 (4,4)	0,292
Marcapasso definitivo	33 (5,4)	3 (3,3)	30 (5,8)	0,608
Insuficiência cardíaca NYHA classes I-II	237 (35,9)	24 (25,3)	213 (37,6)	0,020
Insuficiência cardíaca NYHA classes III-IV	424 (64,1)	71 (74,7)	353 (62,4)	0,020
**Etiologia da doença valvar aórtica**				
Degenerativa	611 (92,4)	88 (92,6)	523 (92,4)	0,193
Reumática	5 (0,8)	1 (1,1)	4 (0,7)	0,601
Congênita	45 (6,8)	6 (6,3)	39 (6,9)	0,999
Endocardite	1 (0,2)	0 (0,0)	1 (0,2)	0,999
**Escore de risco STS**				
Risco baixo	156 (23,6)	9 (9,5)	147 (26,0)	0,001
Risco intermediário	219 (33,1)	25 (26,3)	194 (34,3)	0,127
Risco alto	218 (33,0)	45 (47,4)	173 (30,6)	0,001
Inoperável	68 (10,3)	16 (16,8)	52 (9,2)	0,028

Valores expressos em n (%), média ± desvio padrão. AVE: acidente vascular encefálico; DPOC: doença pulmonar obstrutiva crônica; IAM: infarto agudo do miocárdio; IMC: índice de massa corpórea; ICP: intervenção coronária percutânea; NYHA: New York Heart Association; RM: revascularização miocárdica cirúrgica; STS: Society of Thoracic Surgeons. * Valores de p referem-se à comparação entre Grupo 1 e Grupo 2.

Em procedimentos realizados entre os anos de 2012 e 2017 foi mais frequente o emprego de anestesia geral, de monitorização ecocardiográfica transesofágica, da via de acesso por dissecção e de dispositivos autoexpansíveis. Menor duração do procedimento em minutos e maior taxa de sucesso do implante da bioprótese foram aferidos em implantes efetivados a partir de 2018 (Tabela 2).

**Tabela 2:** – Características dos procedimentos

Variáveis	Total 2012 – 2023 n=661	Grupo 1 2012 – 2017 n=95	Grupo 2 2018 – 2023 n=566	Valor de p*
Estenose aórtica pura	586 (88,7)	81 (85,3)	505 (89,2)	0,293
Insuficiência aórtica pura	4 (0,6)	0 (0,0)	4 (0,7)	0,999
Dupla lesão aórtica	71 (10,7)	14 (14,7)	57 (10,1)	0,208
Eletivo	561 (84,9)	82 (86,3)	479 (84,6)	0,793
Urgência	99 (15,0)	13 (13,7)	86 (15,2)	0,793
Emergência	1 (0,1)	0 (0,0)	1 (0,2)	0,793
Anestesia geral	216 (32,7)	66 (69,5)	150 (26,5)	<0,001
Sedação consciente	445 (67,3)	29 (30,5)	416 (73,5)	<0,001
Epidural	1 (0,2)	0 (0,0)	1 (0,2)	0,999
ETE	203 (30,7)	68 (71,6)	135 (23,9)	<0,001
ETT	458 (68,8)	27 (26,1)	431 (76,2)	<0,001
Proteção cerebral	85 (12,9)	4 (4,2)	81 (14,1)	0,007
Procedimento valve-in-valve	30 (4,5)	2 (2,1)	28 (4,9)	0,292
Conversão para cirurgia cardíaca aberta	4 (0,6)	1 (1,1)	3 (0,5)	0,463
Pré-dilatação	268 (40,5)	24 (25,3)	244 (43,1)	0,001
Pós-dilatação	143 (21,6)	21 (22,1)	122 (21,6)	0,904
Acesso femoral	648 (98,0)	92 (96,8)	556 (98,2)	0,414
**Acesso alternativo**	13 (2,0)	3 (3,2)	10 (1,8)	0,414
Subclávio	9 (1,4)	2 (2,1)	7 (1,2)	
Transcarotídeo	3 (0,5)	0 (0,0)	3 (0,6)	
Transilíaco	1 (0,1)	1 (1,1)	0 (0,0)	
Acesso percutâneo	600 (90,8)	77 (81,1)	523 (92,4)	0,002
Acesso por dissecção	61 (9,2)	18 (18,9)	43 (7,6)	0,002
Balão expansível	306 (46,3)	20 (21,0)	286 (50,5)	<0,001
Mecanicamente expansível	15 (2,3)	15 (15,8)	0 (0,0)	<0,001
Autoexpansível	340 (51,4)	60 (63,2)	280 (49,5)	<0,001
Sucesso do dispositivo	625 (94,6)	85 (89,5)	540 (95,4)	0,018
Duração do procedimento, minutos	118,7 ± 48,5	131,3 ± 49,0	106,1 ± 48,0	<0,001

Valores expressos em n (%), média ± desvio padrão. ETE: ecocardiograma transesofágico; ETT: ecocardiograma transtorácico. *Valores de p referem-se à comparação entre Grupo 1 e Grupo 2.

A taxa de eventos cerebrovasculares (2,6%), infarto agudo miocárdio (1,2%), complicações do acesso vascular de grau importante (3,5%), embolização do dispositivo (0,6%) e cirurgia cardíaca não planejada (0,8%) foi baixa, sem diferenças entre os grupos. A coorte mais contemporânea apresentou menor necessidade de implante de marcapasso definitivo (risco relativo = 0,85, intervalo de confiança de 95% 0,73 a 0,98; p = 0,008), de terapia substitutiva renal (risco relativo = 0,64, intervalo de confiança de 95% 0,36 a 1,15; p = 0,028) e mortalidade (risco relativo = 0,77, intervalo de confiança de 95% 0,60 a 0,98; p = 0,004) durante a fase hospitalar de acompanhamento (Tabela 3).

**Tabela 3:** – Eventos adversos hospitalares

Variáveis	Total 2012 – 2023 n=661	Grupo 1 2012 – 2017 n=95	Grupo 2 2018 – 2023 n=566	Valor de p*
AIT	6 (0,9)	0 (0,0)	6 (1,1)	0,601
AVE isquêmico	10 (1,5)	1 (1,1)	9 (1,6)	0,999
AVE hemorrágico	1 (0,2)	1 (1,1)	0 (0,0)	0,144
IAM	8 (1,2)	0 (0,0)	8 (1,4)	0,610
Intervenção coronária percutânea	7 (1,1)	0 (0,0)	7 (1,2)	0,601
Necessidade de marcapasso definitivo	65 (9,8)	17 (17,9)	48 (8,5)	0,008
Dissecção aórtica	3 (0,5)	0 (0,0)	3 (0,5)	0,999
Perfuração cardíaca	11 (1,7)	3 (3,2)	8 (1,4)	0,201
Migração do dispositivo	4 (0,6)	1 (1,1)	3 (0,5)	0,463
Embolização do dispositivo para VE	2 (0,3)	0 (0,0)	2 (0,4)	0,999
Embolização do dispositivo para aorta	2 (0,3)	1 (1,1)	1 (0,2)	0,267
Reintervenção na valva aórtica	2 (0,3)	0 (0,0)	2 (0,4)	0,999
Cirurgia cardíaca não planejada	5 (0,8)	2 (2,1)	3 (0,5)	0,152
Necessidade de diálise	9 (1,4)	4 (4,2)	5 (0,9)	0,028
Complicação vascular maior	23 (3,5)	5 (5,3)	18 (3,2)	0,357
Complicação vascular menor	17 (2,6)	5 (5,3)	12 (2,1)	0,083
Hematoma na via de acesso	17 (2,6)	2 (2,1)	15 (2,7)	0,999
Cirurgia vascular não planejada	10 (1,5)	2 (2,1)	8 (1,4)	0,643
Sangramento gastrointestinal	5 (0,8)	0 (0,0)	5 (0,9)	0,999
Sangramento urogenital	1 (0,2)	1 (1,1)	1 (0,2)	0,267
Transfusão sanguínea	92 (13,9)	14 (14,7)	78 (13,8)	0,803
Óbito hospitalar	33 (5,0)	11 (11,6)	22 (3,9)	0,004

Valores expressos em n (%). AIT: acidente isquêmico transitório; AVE: acidente vascular encefálico; IAM: infarto agudo do miocárdio;

VE: ventrículo esquerdo. * Valores de p referem-se à comparação entre Grupo 1 e Grupo 2.

## Discussão

Nesta primeira extração de dados do registro TAVIDOR, voltada à evolução temporal observada nas características demográficas, nos procedimentos e nos desfechos hospitalares de pacientes submetidos a TAVI na Rede D’Or São Luiz, constatamos: 1) diminuição da complexidade clínica dos pacientes ao longo dos últimos seis anos, traduzida por maior prevalência de escore de risco STS categorizado como moderado ou baixo e classe funcional I ou II da NYHA, porém sem alteração na média de idade dos pacientes, com predominância de octogenários; 2) incorporação consistente da estratégia de implante minimalista, corroborada pela maior adoção da sedação consciente, da monitorização adjunta pelo ecocardiograma transtorácico e do acesso percutâneo, culminando com menor duração do procedimento; 3) baixa taxa de complicações hospitalares seguindo os critérios VARC-3, com expressiva redução na necessidade de implante de marcapasso definitivo e queda de mortalidade no extrato contemporâneo de pacientes (Figura Central).

Achados semelhantes foram reportados por um registro nacional francês, ao comparar os dados compreendidos entre 2010 e 2012 aos de 2013 a 2015, em um total de 12.489 pacientes submetidos a TAVI.^15^ Constatou-se um menor risco cirúrgico classificado pelo logistic European System for Cardiac Operative Risk Evaluation (EuroSCORE 1) no último período (15,0% versus 18,4%; p < 0,001), queda na utilização de anestesia geral e monitoramento por ecocardiograma transesofágico de 70,3% para 47,2%, e 64,1% para 26,7%, respectivamente, bem como menor mortalidade hospitalar (4,4% versus 8,1%; p < 0,001). Nossos números também vão ao encontro daqueles apresentados por centros latino-americanos participantes do estudo WRITTEN LATAM, cotejando questionários obtidos no ano de 2015 (29 centros) àqueles respondidos entre 2019 e 2020 (46 centros).^16^ De maneira análoga ao Registro TAVIDOR, houve aumento na proporção de pacientes de risco cirúrgico baixo e intermediário tratados com TAVI na América Latina, e na adoção de abordagens minimalistas de 2015 a 2020.

Pacientes do Grupo 2 de nossa casuística apresentaram uma redução de 15% no risco relativo de necessidade de implante de marcapasso definitivo quando comparados aos do Grupo 1. Dois fatores concorreriam para justificar esse resultado. Inicialmente, a mudança no perfil dos dispositivos mais comumente empregados entre os dois períodos, com um aumento significativo no implante de próteses balão-expansíveis, em detrimento das autoexpansíveis e mecanicamente expansíveis. De fato, evidências demonstram maiores taxas de implante de marcapasso definitivo com o uso dos dispositivos Lotus™ (Boston Scientific, Marlborough, MA, EUA) e CoreValve/Evolut R (Medtronic, Minneapolis, MN, EUA), dada a maior compressão provocada por estes no feixe de His, com agravamento de distúrbios de condução prévios e/ou deterioração para bloqueio atrioventricular total.^13,17-19^Entretanto, apesar da queda em sua utilização, próteses autoexpansíveis representaram 49,5% do total atual de procedimentos. Assim, o advento de técnicas de liberação voltadas para uma menor profundidade de implante possivelmente tenha sua parcela de contribuição para a redução alcançada a uma taxa inferior a dois dígitos dessa complicação.^6,20^

Frente aos sucessivos avanços alcançados no tratamento percutâneo da estenose aórtica, culminando com sua indicação no manejo de pacientes categorizados como de baixo risco cirúrgico após a publicação dos estudos seminais PARTNER 3^5^e Evolut Low Risk,^21^ volta-se a atenção à indicação de TAVI em pacientes consequentemente mais jovens, sendo a média de idade nos estudos citados de aproximadamente 74 anos. Embora os dados disponíveis acerca da durabilidade das próteses valvares transcateter ao longo de 5 anos, bem como de sua deterioração estrutural entre 6 e 9 anos pós implante sejam promissores,^22,23^ contemplam pacientes idosos e de alto risco cirúrgico, e devem ser extrapolados com cautela para uma população com maior expectativa de vida e mais propensos a requererem intervenções valvares repetidas. Nesse sentido, o impacto da eventual necessidade de re-acesso coronariano, de novos distúrbios de condução, do implante de marcapasso definitivo, de regurgitações paravalvares, e, em última instância, da indicação de explante de uma prótese transcateter, sabidamente associada a maior mortalidade,^24^devem ser confrontados com a expectativa e preferência do paciente, objetivando o manejo a longo prazo da estenose aórtica. Uma vez que a média de idade da população avaliada no Registro TAVIDOR manteve-se estável ao longo de 10 anos, ao redor de 81 anos, infere-se que a análise crítica da indicação em pacientes mais jovens norteie as decisões do *heart team*.

O estudo exibe limitações: trata-se de um registro de contribuição voluntária, impossibilitando assegurarmos que contemple a totalidade dos procedimentos realizados no período; o preenchimento das informações no banco de dados REDCap não é auditado; ausência de um comitê independente adjudicador de eventos.

## Conclusões

A análise temporal de 10 anos do Registro TAVIDOR demonstra uma queda na complexidade clínica dos pacientes ao longo do tempo, traduzida por maior percentual de pacientes categorizados como de escore de risco cirúrgico baixo ou intermediário, sem que tenha havido mudança na faixa etária, com predominância na abordagem de indivíduos octogenários. Além disso, o natural avanço para técnicas de implante minimalistas, somadas à evolução tecnológica das próteses valvares e seus componentes, podem ter contribuído para menor duração do procedimento, menor necessidade de terapia substitutiva renal, queda na necessidade de implante de marcapasso definitivo e menor taxa de mortalidade hospitalar dentre aqueles cujo implante ocorreu no último quinquênio.

## References

[1] Tarasoutchi F, Montera MW, Ramos AIO, Sampaio RO, Rosa VEE, Accorsi TAD (2020). Update of the Brazilian Guidelines for Valvular Heart Disease - 2020. Arq Bras Cardiol.

[2] Leon MB, Smith CR, Mack M, Miller DC, Moses JW, Svensson LG (2010). Transcatheter Aortic-valve Implantation for Aortic Stenosis in Patients who Cannot Undergo Surgery. N Engl J Med.

[3] Smith CR, Leon MB, Mack MJ, Miller DC, Moses JW, Svensson LG (2011). Transcatheter Versus Surgical Aortic-valve Replacement in High-risk Patients. N Engl J Med.

[4] Leon MB, Smith CR, Mack MJ, Makkar RR, Svensson LG, Kodali SK (2016). Transcatheter or Surgical Aortic-Valve Replacement in Intermediate-Risk Patients. N Engl J Med.

[5] Mack MJ, Leon MB, Thourani VH, Makkar R, Kodali SK, Russo M (2019). Transcatheter Aortic-Valve Replacement with a Balloon-Expandable Valve in Low-Risk Patients. N Engl J Med.

[6] Grubb KJ, Gada H, Mittal S, Nazif T, Rodés-Cabau J, Fraser DGW (2023). Clinical Impact of Standardized TAVR Technique and Care Pathway: Insights From the Optimize PRO Study. JACC Cardiovasc Interv.

[7] Cribier A, Eltchaninoff H, Bash A, Borenstein N, Tron C, Bauer F (2002). Percutaneous Transcatheter Implantation of an Aortic Valve Prosthesis for Calcific Aortic Stenosis: First Human Case Description. Circulation.

[8] Grube E, Laborde JC, Zickmann B, Gerckens U, Felderhoff T, Sauren B (2005). First Report on a Human Percutaneous Transluminal Implantation of a Self-expanding Valve Prosthesis for Interventional Treatment of Aortic Valve Stenosis. Catheter Cardiovasc Interv.

[9] Cribier A, Eltchaninoff H, Tron C, Bauer F, Agatiello C, Nercolini D (2006). Treatment of Calcific Aortic Stenosis with the Percutaneous Heart Valve: Mid-term Follow-up from the Initial Feasibility Studies: The French Experience. J Am Coll Cardiol.

[10] Grube E, Schuler G, Buellesfeld L, Gerckens U, Linke A, Wenaweser P (2007). Percutaneous Aortic Valve Replacement for Severe Aortic Stenosis in High-risk Patients Using the Second- and Current Third-generation Self-expanding CoreValve Prosthesis: Device Success and 30-day Clinical Outcome. J Am Coll Cardiol.

[11] Perin MA, Brito FS, Almeida BO, Pereira MA, Abizaid A, Tarasoutchi F (2009). Percutaneous Aortic Valve Replacement for the Treatment of Aortic Stenosis: Early Experience in Brazil. Arq Bras Cardiol.

[12] Silva LS, Caramori PR, Nunes AC, Katz M, Guaragna JC, Lemos P (2015). Performance of Surgical Risk Scores to Predict Mortality after Transcatheter Aortic Valve Implantation. Arq Bras Cardiol.

[13] Monteiro C, Ferrari ADL, Caramori PRA, Carvalho LAF, Siqueira DAA, Thiago LEKS (2017). Permanent Pacing after Transcatheter Aortic Valve Implantation: Incidence, Predictors and Evolution of Left Ventricular Function. Arq Bras Cardiol.

[14] Généreux P, Piazza N, Alu MC, Nazif T, Hahn RT, Pibarot P (2021). Valve Academic Research Consortium 3: Updated Endpoint Definitions for Aortic Valve Clinical Research. Eur Heart J.

[15] Auffret V, Lefevre T, Van Belle E, Eltchaninoff H, Iung B, Koning R (2017). Temporal Trends in Transcatheter Aortic Valve Replacement in France: FRANCE 2 to FRANCE TAVI. J Am Coll Cardiol.

[16] Bernardi FLM, Ribeiro HB, Nombela-Franco L, Cerrato E, Maluenda G, Nazif T (2022). Recent Developments and Current Status of Transcatheter Aortic Valve Replacement Practice in Latin America - the WRITTEN LATAM Study. Arq Bras Cardiol.

[17] Gensas CS, Caixeta A, Siqueira D, Carvalho LA, Sarmento-Leite R, Mangione JA (2014). Predictors of Permanent Pacemaker Requirement after Transcatheter Aortic Valve Implantation: Insights from a Brazilian Registry. Int J Cardiol.

[18] Costa G, Barbanti M, Rosato S, Seccareccia F, Tarantini G, Fineschi M (2022). Real-World Multiple Comparison of Transcatheter Aortic Valves: Insights From the Multicenter OBSERVANT II Study. Circ Cardiovasc Interv.

[19] Reardon MJ, Feldman TE, Meduri CU, Makkar RR, O'Hair D, Linke A (2019). Two-Year Outcomes after Transcatheter Aortic Valve Replacement With Mechanical vs Self-expanding Valves: The REPRISE III Randomized Clinical Trial. JAMA Cardiol.

[20] Yoon SH, Galo J, Amoah JK, Dallan LAP, Tsushima T, Motairek IK (2023). Permanent Pacemaker Insertion Reduction and Optimized Temporary Pacemaker Management after Contemporary Transcatheter Aortic Valve Implantation With Self-Expanding Valves (from the Pristine TAVI Study). Am J Cardiol.

[21] Popma JJ, Deeb GM, Yakubov SJ, Mumtaz M, Gada H, O'Hair D (2019). Transcatheter Aortic-Valve Replacement with a Self-Expanding Valve in Low-Risk Patients. N Engl J Med.

[22] Blackman DJ, Saraf S, MacCarthy PA, Myat A, Anderson SG, Malkin CJ (2019). Long-Term Durability of Transcatheter Aortic Valve Prostheses. J Am Coll Cardiol.

[23] Søndergaard L, Ihlemann N, Capodanno D, Jørgensen TH, Nissen H, Kjeldsen BJ (2019). Durability of Transcatheter and Surgical Bioprosthetic Aortic Valves in Patients at Lower Surgical Risk. J Am Coll Cardiol.

[24] Hawkins RB, Deeb GM, Sukul D, Patel HJ, Gualano SK, Chetcuti SJ (2023). Redo Surgical Aortic Valve Replacement after Prior Transcatheter Versus Surgical Aortic Valve Replacement. JACC Cardiovasc Interv.

