# Supplementary material for: Tendência Temporal no Implante Percutâneo de Bioprótese Aórtica: Análise de 10 Anos do Registro TAVIDOR
Source: Arq Bras Cardiol. 2024 May 23;121(5):e20230467. [Article in Portuguese] doi: 10.36660/abc.20230467 (PMC11164433; doi:10.36660/abc.20230467)
Supplement: Translation

# Translation: Temporal Trends in Transcatheter Aortic Valve Implantation: 10-Year Analysis of the TAVIDOR Registry

**DOI:** 10.36660/abc.202300467i

**Authors:** Vinicius Esteves, Pedro Beraldo de Andrade, Cleverson Neves Zukowski, Edmur Araujo, Cristiano Guedes Bezerra, Adriano Dourado Oliveira, Eduardo Pessoa de Melo, Gustavo Gama, Rodrigo Cantarelli, Luiz Alberto Piva e Mattos, Angelo Tedeschi, Vitor Alves Loures, Vitor Vahle, Guilherme Barreto Gameiro Silva, Miguel Antonio Neves Rati, Augusto Celso Lopes, Nilson de Moura Fé, Gustavo Alves, Sérgio Costa Tavares, Sergio Kreimer, Marden Tebet, Felipe Maia, Maurício Sales de Oliveira, Alberto Fonseca, Angelina Camiletti, Denilson Campos de Albuquerque, Olga Ferreira de Souza

**Affiliations:** 1 Brazil Rede D’Or São Luiz, São Paulo, SP – Brazil; 2 Brazil Santa Casa de Misericórdia de Marília – Cardiologia Invasiva, Marília, SP – Brazil; 3 Brazil Hospital Copa D’Or, Rio de Janeiro, RJ – Brazil; 4 Brazil Hospital Quinta D’Or, Rio de Janeiro, RJ – Brazil; 5 Brazil Hospital do Coração do Brasil, Brasília, RJ – Brazil; 6 Brazil Universidade Federal da Bahia – Hemodinâmica e Cardiologia Intervencionista, Salvador, BA – Brazil; 7 Brazil Hospital Santa Izabel, Salvador, BA – Brazil; 8 Brazil Hospital Esperança, Recife, PE – Brazil; 9 Brazil UDI Hospital, São Luís, MA – Brazil; 10 Brazil Hospital Memorial São José, Recife, PE – Brazil; 11 Brazil Rede D’Or São Luiz, Rio de Janeiro, RJ – Brazil; 12 Brazil Hospital São Luiz Anália Franco, São Paulo, SP – Brazil; 13 Brasil Hospital São Lucas, Aracaju, SE – Brasil; 14 Brazil Hospital Santa Cruz, Curitiba, PR – Brazil; 15 Brazil Hospital Barra D’Or, Rio de Janeiro, RJ – Brazil; 16 Brazil Hospital Monte Klinikum, Fortaleza, CE – Brazil; 17 Brazil Hospital São Carlos, Fortaleza, CE – Brazil; 18 Brazil Hospital e Maternidade Brasil, Santo André, SP – Brazil; 19 Brazil Universidade do Estado do Rio de Janeiro, Rio de Janeiro, RJ – Brazil

**Keywords:** Aortic Valve Stenosis, Transcatheter Aortic Valve Replacement, Spatio-Temporal Analysis, Aged

## Abstract

**Background:**

Transcatheter aortic valve implantation (TAVI) has established itself as the preferential strategy to approach severe aortic stenosis. Information on procedural improvements and nationwide results obtained with the technique throughout the past decade are unknown.

**Objectives:**

To assess the temporal variation of the demographic profile, procedural characteristics, and in-hospital outcomes of patients undergoing TAVI procedures at the Rede D’Or São Luiz.

**Methods:**

Observational registry comprising 29 national institutions, comparing the characteristics of the TAVI procedures performed from 2012 to 2017 (Group 1) to those performed from 2018 to 2023 (Group 2). The statistical significance level adopted was p < 0.05.

**Results:**

This study assessed 661 patients, 95 in Group 1 and 566 in Group 2, with a mean age of 81.1 years. Group 1 patients had a higher prevalence of New York Heart Association functional class III or IV and STS risk score > 8%. In addition, they more often underwent general anesthesia, transesophageal echocardiographic monitoring, and access through femoral dissection. Group 2 patients had a higher success rate of the TAVI procedure (95.4% versus 89.5%; p = 0.018), lower mortality (3.9% versus 11.6%; p = 0.004), and less often needed permanent pacemaker implantation (8.5% versus 17.9%; p = 0.008).

**Conclusions:**

The 10-year temporal trends analysis of the TAVIDOR Registry shows a reduction in patients’ clinical complexity over time. Furthermore, the advance to minimalistic implantation techniques, added to the technological evolution of the devices, may have contributed to the favorable outcomes observed among those whose implantation occurred in the last 5 years studied.

## Introduction

Over time, transcatheter aortic valve implantation (TAVI) has established itself as the preferential strategy to approach severe symptomatic aortic stenosis in patients aged 70 years and older, those deemed inoperable, or those with contraindications to conventional surgery or significant frailty.^1^

In the successful trajectory of this therapeutic modality, it was paramount to initially assess the efficacy and safety of first-generation devices in inoperable patients.^2^ The subsequent use of the technique in individuals with a less complex profile occurred simultaneously with the advances in the prostheses’ design, such as the incorporation of the outer skirt seal, the possibility of recapture, the reduction in the introducers’ profile and caliber, as well as the improvement in the implantation technique, which resulted in lower rates of paravalvular regurgitation, need for permanent pacemaker, stroke, and vascular complications.^3-6^

After the first description of TAVI in human beings with a balloon-expandable device in 2002 by Cribier et al.^7^ and with a self-expanding prosthesis in 2005 by Grube et al.,^8^ international registries began to report the use of the technique in obtaining favorable results of efficacy and safety.^9,10^The initial experience in Brazil dates back to 2008,^11^ and, since then, data on national results have been obtained from publications generated from the analysis of the Brazilian Registry of Transcatheter Aortic Valve Implantation, a multicenter registry, in which participation is voluntary, managed by the Sociedade Brasileira de Hemodinâmica e Cardiologia Intervencionista.^12,13^ However, information on the patients’ clinical outcomes and procedural improvements, as well as the nationwide results obtained with the technique throughout the past decade, are unknown.

## Objectives

To assess the temporal trends of the demographic profile, procedural characteristics, and in-hospital outcomes of patients undergoing TAVI procedures by comparing data from patients treated from 2012 to 2017 to those of patients treated from 2018 to May 2023, with an analysis of comparable time intervals between two groups.

## Methods

### Study design and population

This was an observational, multicenter registry comprising 29 Brazilian institutions in the states of São Paulo (9), Rio de Janeiro (7), Pernambuco (4), Bahia (3), Ceará (1), Maranhão (1), Sergipe (1), Paraná (1), and Distrito Federal (2). The study retrospectively included patients who had undergone TAVI from August 2012 to December 2019 and prospectively included patients undergoing TAVI from January 2020 on.

The study sample consisted of patients with severe aortic stenosis, aged ≥ 60 years, submitted to TAVI after providing written informed consent. Patients not submitted to angiotomographic assessment prior to the procedure were excluded from the registry.

The registry was approved by the Committee on Ethics and Research of the Instituto D’Or de Pesquisa e Ensino, and it followed the recommendations of the World Health Organization, the Helsinki Declaration, and the National Health Council’s Resolution 466/2012.

### Study procedures

The decision to perform TAVI and the choice of the device to be used were made by a multidisciplinary team involving a clinical cardiologist, an interventional cardiologist, and a heart surgeon (heart team). The procedures inherent in implantation and care up to hospital discharge followed the institutional routine of each center participating in the registry. The TAVIDOR Registry protocol requires follow-up by use of telephone contact, electronic contact (e-mail), or in-person outpatient visits at 30 days, 6 and 12 months, and then annually for 5 years, when the patients or family members are asked about symptoms, medications used, laboratory tests, and clinical outcomes, such as hospitalizations and events as defined in the Valve Academic Research Consortium 3 (VARC-3) criteria.^14^ The criterion adopted for dividing the patients into two groups was the creation of two population strata over approximately 5 years of observation each.

### Statistical analysis

Data were extracted from the REDCap platform, used by the centers for online input of information on patients and procedures. The statistical analysis was performed with SPSS software, version 17.0 (SPSS Inc., Chicago, IL, USA). Categorical variables were described as frequency, while continuous variables were described as mean and standard deviation or median and interquartile range, according to their distribution pattern, assessed by the Kolmogorov-Smirnov test. In univariate analysis, categorical variables were compared using the chi-square test, while continuous variables were compared using Student’s t test. The statistical significance level of p < 0.05 was adopted.

## Results

Figure 1 shows the flowchart of patients submitted to the TAVI procedure in this analysis. From August 2012 to May 2023, 661 procedures were performed, 95 up to December 2017 (Group 1) and 566 from January 2018 on (Group 2).

**Figure 1:** – Flowchart of the temporal trends of patients undergoing percutaneous implantation of aortic valve bioprosthesis at Rede D’Or São Luiz.

The patients’ mean age was 81.1 years, and the degenerative etiology of heart valve disease predominated (92.4%). There was a high prevalence of concomitant atherosclerotic coronary disease (38.5%), chronic renal failure (30.7%), and peripheral artery disease (24.7%). Group 1 had a higher percentage of women, New York Heart Association (NYHA) functional class III or IV heart failure, and Society of Thoracic Surgeons (STS) risk score > 8% as compared to Group 2 (Table 1).

**Table 1:** – Baseline clinical characteristics of the registry’s general population according to period of inclusion

Clinical characteristics	Total 2012 – 2023 n=661	Group 1 2012 – 2017 n=95	Group 2 2018 – 2023 n=566	p value*
Female sex	324 (49.0)	57 (60.0)	267 (47.2)	0.022
Male sex	337 (51.0)	38 (40.0)	299 (52.8)	0.022
Age, years	81.1 ± 7.1	81.3 ± 6.6	81.0 ± 7.6	0.748
Arterial hypertension	560 (84.7)	78 (82.1)	482 (85.2)	0.444
Diabetes mellitus	234 (35.4)	33 (34.7)	201 (35.5)	0.884
Dyslipidemia	412 (62.3)	52 (54.7)	360 (63.6)	0.099
Smoking	36 (5.4)	2 (2.1)	34 (6.0)	0.121
Chronic renal failure	203 (30.7)	28 (29.5)	175 (30.9)	0.778
Hemodialysis	18 (2.7)	1 (1.1)	17 (3.0)	0.280
Peripheral artery disease	163 (24.7)	24 (25.3)	139 (24.6)	0.883
Carotid artery disease	35 (5.3)	3 (3.2)	32 (5.7)	0.315
COPD	98 (14.8)	15 (15.8)	83 (14.7)	0.775
Atrial fibrillation	125 (20.0)	12 (13.0)	113 (21.2)	0.118
Previous AMI	91 (13.8)	13 (13.7)	78 (13.8)	0.980
Previous stroke	44 (6.7)	4 (4.2)	40 (7.1)	0.301
Previous PCI	184 (27.8)	23 (24.2)	161 (28.4)	0.394
Previous CABG	71 (10.7)	14 (14.7)	57 (10.1)	0.174
Previous aortic valve replacement	27 (4.1)	2 (2.1)	25 (4.4)	0.292
Permanent pacemaker	33 (5.4)	3 (3.3)	30 (5.8)	0.608
NYHA class I-II heart failure	237 (35.9)	24 (25.3)	213 (37.6)	0.020
NYHA class III-IV heart failure	424 (64.1)	71 (74.7)	353 (62.4)	0.020
**Aortic valve disease etiology**				
Degenerative	611 (92.4)	88 (92.6)	523 (92.4)	0.193
Rheumatic	5 (0.8)	1 (1.1)	4 (0.7)	0.601
Congenital	45 (6.8)	6 (6.3)	39 (6.9)	0.999
Endocarditis	1 (0.2)	0 (0.0)	1 (0.2)	0.999
**STS risk score**				
Low	156 (23.6)	9 (9.5)	147 (26.0)	0.001
Intermediate	219 (33.1)	25 (26.3)	194 (34.3)	0.127
High	218 (33.0)	45 (47.4)	173 (30.6)	0.001
Inoperable	68 (10.3)	16 (16.8)	52 (9.2)	0.028

Values expressed as n (%), mean ± standard deviation. AMI: acute myocardial infarction; BMI: body mass index; CABG: coronary artery bypass grafting; COPD: chronic obstructive pulmonary disease; NYHA: New York Heart Association; PCI: percutaneous coronary intervention; STS: Society of Thoracic Surgeons. *P values refer to comparison between Group 1 and Group 2.

In procedures performed from 2012 to 2017, general anesthesia, transesophageal echocardiographic monitoring, access through dissection, and self-expanding devices were more frequently used. Shorter duration (in minutes) and higher success rate of the TAVI procedure were observed from 2018 on (Table 2).

**Table 2:** – Characteristics of the transcatheter aortic valve implantation procedures

Variables	Total 2012 – 2023 n=661	Group 1 2012 – 2017 n=95	Group 2 2018 – 2023 n=566	p value*
Pure aortic stenosis	586 (88.7)	81 (85.3)	505 (89.2)	0.293
Pure aortic insufficiency	4 (0.6)	0 (0.0)	4 (0.7)	0.999
Double aortic lesion	71 (10.7)	14 (14.7)	57 (10.1)	0.208
Elective	561 (84.9)	82 (86.3)	479 (84.6)	0.793
Urgent	99 (15.0)	13 (13.7)	86 (15.2)	0.793
Emergency	1 (0.1)	0 (0.0)	1 (0.2)	0.793
General anesthesia	216 (32.7)	66 (69.5)	150 (26.5)	<0.001
Conscious sedation	445 (67.3)	29 (30.5)	416 (73.5)	<0.001
Epidural	1 (0.2)	0 (0.0)	1 (0.2)	0.999
TEE	203 (30.7)	68 (71.6)	135 (23.9)	<0.001
TTE	458 (68.8)	27 (26.1)	431 (76.2)	<0.001
Cerebral protection	85 (12.9)	4 (4.2)	81 (14.1)	0.007
Valve-in-valve procedure	30 (4.5)	2 (2.1)	28 (4.9)	0.292
Conversion to open cardiac surgery	4 (0.6)	1 (1.1)	3 (0.5)	0.463
Pre-dilatation	268 (40.5)	24 (25.3)	244 (43.1)	0.001
Post-dilatation	143 (21.6)	21 (22.1)	122 (21.6)	0.904
Femoral access	648 (98.0)	92 (96.8)	556 (98.2)	0.414
**Alternative access**	13 (2.0)	3 (3.2)	10 (1.8)	0.414
Subclavian	9 (1.4)	2 (2.1)	7 (1.2)	
Transcarotid	3 (0.5)	0 (0.0)	3 (0.6)	
Transiliac	1 (0.1)	1 (1.1)	0 (0.0)	
Percutaneous access	600 (90.8)	77 (81.1)	523 (92.4)	0.002
Access through dissection	61 (9.2)	18 (18.9)	43 (7.6)	0.002
Balloon-expandable	306 (46.3)	20 (21.0)	286 (50.5)	<0.001
Mechanically expandable	15 (2.3)	15 (15.8)	0 (0.0)	<0.001
Self-expanding	340 (51.4)	60 (63.2)	280 (49.5)	<0.001
Successful procedure	625 (94.6)	85 (89.5)	540 (95.4)	0.018
Procedure’s duration, minutes	118.7 ± 48.5	131.3 ± 49.0	106.1 ± 48.0	<0.001

Values expressed as n (%), mean ± standard deviation. TEE: transesophageal echocardiogram; TTE: transthoracic echocardiogram. * P values refer to comparison between Group 1 and Group 2.

The rates of cerebrovascular events (2.6%), acute myocardial infarction (1.2%), significant vascular complications (3.5%), device embolization (0.6%), and unplanned cardiac surgery (0.8%) were low and showed no difference between the groups. Group 2 patients less often needed permanent pacemaker implantation (relative risk = 0.85; 95% confidence interval, 0.73 to 0.98; p = 0.008) and renal replacement therapy (relative risk = 0.64; 95% confidence interval, 0.36 to 1.15; p = 0.028), and had lower mortality (relative risk = 0.77; 95% confidence interval, 0.60 to 0.98; p = 0.004) during hospitalization (Table 3).

**Table 3:** – In-hospital adverse events

Variables	Total 2012 – 2023 n=661	Group 1 2012 – 2017 n=95	Group 2 2018 – 2023 n=566	p value*
TIA	6 (0,9)	0 (0,0)	6 (1,1)	0,601
Ischemic stroke	10 (1,5)	1 (1,1)	9 (1,6)	0,999
Hemorrhagic stroke	1 (0,2)	1 (1,1)	0 (0,0)	0,144
AMI	8 (1,2)	0 (0,0)	8 (1,4)	0,610
Percutaneous coronary intervention	7 (1,1)	0 (0,0)	7 (1,2)	0,601
Need for permanent pacemaker	65 (9,8)	17 (17,9)	48 (8,5)	0,008
Aortic dissection	3 (0,5)	0 (0,0)	3 (0,5)	0,999
Cardiac perforation	11 (1,7)	3 (3,2)	8 (1,4)	0,201
Device migration	4 (0,6)	1 (1,1)	3 (0,5)	0,463
Device embolization to the LV	2 (0,3)	0 (0,0)	2 (0,4)	0,999
Device embolization to the aorta	2 (0,3)	1 (1,1)	1 (0,2)	0,267
Reintervention in the aortic valve	2 (0,3)	0 (0,0)	2 (0,4)	0,999
Unplanned cardiac surgery	5 (0,8)	2 (2,1)	3 (0,5)	0,152
Need for dialysis	9 (1,4)	4 (4,2)	5 (0,9)	0,028
Major vascular complication	23 (3,5)	5 (5,3)	18 (3,2)	0,357
Minor vascular complication	17 (2,6)	5 (5,3)	12 (2,1)	0,083
Access site hematoma	17 (2,6)	2 (2,1)	15 (2,7)	0,999
Unplanned vascular surgery	10 (1,5)	2 (2,1)	8 (1,4)	0,643
Gastrointestinal bleeding	5 (0,8)	0 (0,0)	5 (0,9)	0,999
Urogenital bleeding	1 (0,2)	1 (1,1)	1 (0,2)	0,267
Blood transfusion	92 (13,9)	14 (14,7)	78 (13,8)	0,803
In-hospital death	33 (5,0)	11 (11,6)	22 (3,9)	0,004

Values expressed as n (%). AMI: acute myocardial infarction; LV: left ventricle; TIA: transient ischemic attack. *P values refer to comparison between Group 1 and Group 2.

## Discussion

In this first data extraction from the TAVIDOR Registry, regarding the temporal variation of the demographic and procedural characteristics and in-hospital outcomes of patients undergoing TAVI procedure at the Rede D’Or São Luiz, we observed: 1) a reduction in the clinical complexity of the patients over the last six years of the study, evidenced by the higher prevalence of the STS risk score categorized as moderate or low and NYHA functional class I or II, but no change in the patients’ mean age, with predominance of octogenarians; 2) consistent incorporation of the minimalist approach strategy, corroborated by the more frequent adoption of conscious sedation, adjunct transthoracic echocardiogram monitoring, and percutaneous access, culminating in shorter procedure duration; 3) low rate of in-hospital complications as defined by the VARC-3 criteria, with a significant reduction in the need for permanent pacemaker implantation and in mortality in Group 2 patients (Central Illustration).

Similar findings have been reported in a French national registry comparing data from the periods 2010 to 2012 and 2013 to 2015, in a total of 12,489 patients undergoing TAVI procedure.^15^ The authors observed, in the latter period, lower surgical risk classified according to the logistic European System for Cardiac Operative Risk Evaluation (EuroSCORE 1) (15.0% versus 18.4%; p < 0.001), a decrease in the use of general anesthesia and of transesophageal echocardiographic monitoring (from 70.3% to 47.2% and from 64.1% to 26.7%, respectively), in addition to lower in-hospital mortality (4.4% versus 8.1%; p < 0.001). Our numbers are in accordance with those reported by Latin-American centers participating in the WRITTEN LATAM study, which compared questionnaires obtained in 2015 (29 centers) with questionnaires answered from 2019 to 2020 (46 centers).^16^ Similarly to the findings in the TAVIDOR Registry, there was an increase in the proportion of patients with low and intermediate surgical risk treated with TAVI in Latin America, as well as in the adoption of minimalist approach from 2015 to 2020.

In our study, Group 2 patients showed a 15% reduction in the relative risk of need for permanent pacemaker implantation as compared to Group 1 patients. Two factors could explain that result. The first is the change in the profile of the devices most frequently used in the two periods, with a significant increase in the implantation of balloon-expandable prostheses instead of self-expanding and mechanically expanded prostheses. In fact, there is evidence of higher rates of permanent pacemaker implantation with the use of the Lotus™ (Boston Scientific, Marlborough, MA, USA) and CoreValve/Evolut R (Medtronic, Minneapolis, MN, USA) devices, because of higher compression of the His bundle, with worsening of previous conduction disorders and/or deterioration to complete atrioventricular block.^13,17-19^However, despite the reduction in their use, self-expanding prostheses currently account for 49.5% of all procedures. Thus, the emergence of current techniques designed for shallower implantation depth might have contributed to the reduction in that complication to rates under two digits.^6,20^

Given the successive improvements in the percutaneous treatment of aortic stenosis, culminating in its indication for the management of patients categorized as at low surgical risk after the publication of the seminal studies PARTNER 3^5^and Evolut Low Risk,^21^ attention has been given to the indication of TAVI for younger patients, the mean age in those studies being around 74 years. Although data on the 5-year durability of transcatheter valve prostheses and their structural deterioration in 6 to 9 years after implantation are promissing,^22,23^ they were obtained from elderly and high-surgical-risk patients and should, therefore, be carefully extrapolated to a population with longer life expectancy prone to require repeat valve interventions. The impact of the occasional need for coronary re-access, of new conduction disorders, of permanent pacemaker implantation, of paravalvular regurgitation, and, eventually, of the indication of transcatheter prosthesis explantation, known to be associated with higher mortality,^24^should be weighed against the patient’s expectation and preference, aiming at the long-term management of aortic stenosis. Considering that the mean age of the TAVIDOR Registry population, around 81 years, remained stable over 10 years, we infer that the critical analysis of the procedure indication for younger patients guides the heart team’s decisions.

Some limitations of this study are as follows: participation in the TAVIDOR Registry is voluntary; thus, one cannot guarantee that it contemplates all procedures performed in the period; the input of information into the REDCap platform is not audited; and lack of an independent event adjudication committee.

## Conclusions

The 10-year temporal analysis of the TAVIDOR Registry shows a reduction in the clinical complexity of the patients over time, represented by a higher percentage of patients categorized as at low or intermediate surgical risk, with no change in their age range and predominance of octogenarians. Furthermore, the advance to implantation techniques with a minimalist approach, added to the technological evolution of the valve prostheses and their components, may have contributed to shorter procedure duration, reduced need for renal replacement therapy and permanent pacemaker implantation, and reduced in-hospital mortality among those who underwent prosthesis implantation in the last 5 years studied.
